# A comprehensive analysis of the Korean fir (*Abies koreana*) genes expressed under heat stress using transcriptome analysis

**DOI:** 10.1038/s41598-018-28552-1

**Published:** 2018-07-06

**Authors:** Jung Eun Hwang, Yun Jeong Kim, Myung Hwan Shin, Hwa Ja Hyun, Hans J. Bohnert, Hyeong Cheol Park

**Affiliations:** 1 Division of Ecological Conservation, Bureau of Ecological Research, National Institute of Ecology, Seocheon, Republic of Korea; 2 National Institute Forest Science Warm Temperate and Subtropical Forest Research Center, Jeju, Republic of Korea; 3 Department of Plant Biology, University of Illinois at Urbana-Champaign, Urbana, IL 61801 USA

## Abstract

Korean fir (*Abies koreana*), a rare species endemic to South Korea, is sensitive to climate change. Here, we used next-generation massively parallel sequencing technology and *de novo* transcriptome assembly to gain a comprehensive overview of the Korean fir transcriptome under heat stress. Sequencing control and heat-treated samples of Korean fir, we obtained more than 194,872,650 clean reads from each sample. After *de novo* assembly and quantitative assessment, 42,056 unigenes were generated with an average length of 908 bp. In total, 6,401 differentially expressed genes were detected, of which 2,958 were up-regulated and 3,443 down-regulated, between the heat-treated and control samples. A gene ontology analysis of these unigenes revealed heat-stress-related terms, such as “response to stimulus”. Further, in depth analysis revealed 204 transcription factors and 189 Hsps as differentially expressed. Finally, 12 regulated candidate genes associated with heat stress were examined using quantitative real-time PCR (qRT–PCR). In this study, we present the first comprehensive characterisation of Korean fir subjected to heat stress using transcriptome analysis. It provides an important resource for future studies of Korean fir with the objective of identifying heat stress tolerant lines.

## Introduction

Korean fir (*Abies koreana*), a valuable tree species for ornamental purposes, is an endemic and rare species in Korea^1^. It grows in the upper regions of Mounts Halla, Chiri, Mudung, Kaji and Dukyu, which are located in the southern part of the Korean peninsula^1^. Recently, the Korean fir population underwent a large dieback, resulting in a severe decline. This dieback may have been the result of complex interactions among multiple environmental factors caused by global warming^2^. Because this species is susceptible to climate changes^2,3^, it was designated an indicator species illustrating climate change by the Korean government^4^. The resistance to high temperature has been investigated in few tree species, including *Acer rubrum*^5^, *Picea glauca*^6^. For most trees, tolerance to high temperatures remains largely unstudied. Thus, it is essential to uncover and understand molecular response mechanisms of the Korean fir to heat stress, with the objective of gaining an understanding of mechanisms that impede heat tolerance in this species.

High temperatures can reduce plant growth and development, which may become a major issue in the coming years owing to global warming^7^. Global temperatures are predicted to rise by an additional at least 2 °C, and possibly more, by the end of this century. Therefore, a better understanding of plant heat tolerance mechanisms is crucial for the sustainable preservation of forest. Plants respond to high temperatures by altering the expression levels of thousands of genes, thereby changing cellular, physiological, and biochemical processes. When experiencing stress, the expression of a number genes changes, enabling plants to respond to the abiotic stressor and consequently to control or mitigate deleterious effects. Several transcriptional regulatory networks are involved in stress-induced changes in gene expression^8^. In a hierarchical mode, stress-induced genes can up-regulate the expression levels of many downstream genes that then may provide abiotic stress tolerance to extreme temperatures, drought, or high salinity^9,10^. These include various transcription factors (TFs) such as dehydration responsive element-binding (DREB) and heat shock transcription factors (HSFs), suggesting the roles of various transcriptional regulatory mechanisms in the stress-signal transduction pathways^9^. These TFs regulate the expression of heat shock proteins (HSPs), which play a central role in the heat-stress response^11^. The tolerance conferred by HSPs then reinforces physiological functions, such as photosynthesis, assimilate partitioning, water and nutrient use efficiency levels, and membrane stability^7^. However, there are differences in responses to heat stress among different species and often also genotypes of one species^12^. For the vast majority of species, their transcriptomes are even now largely uncharacterised, and even in species for which substantial information is available, it is often in the form of partially characterized transcriptomes only.

The analysis of gene expression levels is a valuable tool in understanding the transcriptome dynamics and the potential for manipulating gene expression patterns in plants. The development of a high-throughput sequencing technology, RNA sequencing (RNA-seq), has been successfully used for gene expression profiling and other transcriptome studies in an increasing number of plants, including Arabidopsis^13^, rice^14^, and poplar^15^. In the past, the *de novo* assembly of very short-read sequences without a known reference posed often severe difficulties^16^. However, the recent development and optimisation of a *de novo* short-read assembly methods now allows for rapid and cost-effective assembly of transcriptomes also of non-model organisms with unknown genomes, opening the door for performing numerous and substantial new analysis opportunities^17,18^. This method made possible sequencing of transcriptomes in species not previously approachable, such as *Picea abies*^19,20^. However, no comparative transcriptomic analyses have been performed using next-generation sequencing technologies in the genus *Abies* under an environmental stress simulation.

In our study, the genome-wide analysis of gene expression levels during a heat treatment was performed with Korean fir in the genus *Abies* using a next generation sequencing-based Illumina paired-end platform. The data were used to create a reference transcriptome, containing an extensive set of genes expressed under heat treatment. Moreover, we sought and identified genes with important roles in heat tolerance. The results will provide understanding of the adaptive heat-stress mechanisms in Korean fir at the molecular level, and increase the genomic information available for the genus *Abies*.

## Results

### Transcriptome sequencing and *de novo* assembly

To elucidate the molecular responses to heat stress in Korean fir, we prepared six libraries from heat-treated and control samples for sequencing. In total, 1,330,756,430 raw reads (ranging from 196,808,520 to 237,953,246 for each sample) were obtained from the control and heat-treated samples (Table 1). From these, 236,917,754; 207,072,292; 227,273,940; 233,574,546; 194,872,650 and 223,806,200 clean reads were obtained from each sample, respectively (Table 1). Of the clean reads, the Q20 percentage (sequencing error rate < 1%) was over 95% and the G + C content was approximately 45% for all libraries (Table 1). The six libraries reads were combined to assembly the Korean fir transcriptome. Transcriptome *de novo* assembly was performed using Trinity software, which generated 406,207 transcripts with average length of 472.74 bp and an N50 of 532 bp for the merged assembly of six libraries (Table 2). The N50 value improved to 1,608 bp after running CD-HIT and filtered raw read count with total number of transcripts reduced to 42,056 (Table 2). The high-quality reads produced in this study have been deposited in the NCBI SRA database (accession number: SRR6781816, SRR6781817, SRR6781818, SRR6781819, SRR6781820 and SRR6781821).

**Table 1:** Quality of Korean fir’s sequencing.

Sample	Raw reads	Clean reads	Clean bases	GC (%)	Q20 (%)	Q30 (%)
Control_1	237,953,246	236,917,754	23,844,037,594	44.28	95.61	97.52
Control_2	207,999,230	207,072,292	20,854,653,490	44.33	95.42	97.44
Control_3	228,337,054	227,273,940	22,875,259,422	44.99	95.41	97.43
Heat-treated_1	234,835,376	233,574,546	23,512,736,318	45.22	95.48	97.47
Heat-treated_2	196,808,520	194,872,650	19,606,902,313	45.83	95.16	97.25
Heat-treated_3	224,823,004	223,806,200	22,542,923,111	45.31	95.52	97.48

**Table 2:** Length distributions of the assembled *Abies koreana* transcripts.

Type	Contigs	Unigenes
Total trinity transcripts	406,207	42,056
Minimum length (bp)	201	201
Maximum length (bp)	19,314	19,314
Average length (bp)	472.74	908
N50 (bp)	532	1,608
Total length (bp)	192,031,706	51,118,088

### Functional annotation and classification of the Korean fir transcriptome

**Figure 1:**
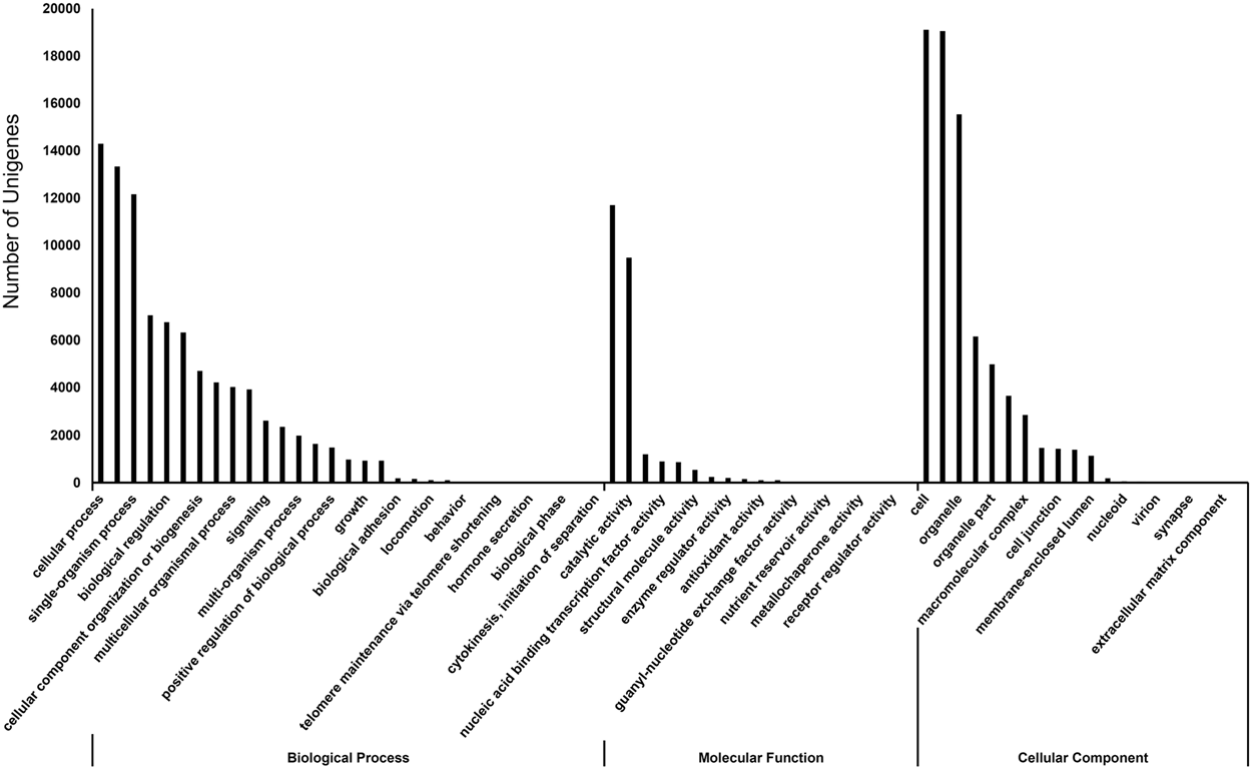
Gene Ontology (GO) classification of *Abies koreana* unigenes. A total of 42,056 unigenes were functionally classified into three main functional categories: biological processes, molecular functions, and cellular components.

For annotation purposes, the 42,056 assembled unigenes were analysed for gene ontology (GO) terms using gene ontology database and WEGO. Altogether, 22,880 unigenes, 54.40% of the total unigenes, were annotated using the GO database. The annotated Korean fir genes were functionally categorised based on the GO classification system, which contains three major functional categories, biological processes, molecular functions, and cellular components (Supplemental Table S1 and Fig. 1). For the category of biological process, the most abundant groups were “cellular process” (14,297 genes), “metabolic process” (13,336 genes), “single-organism process” (12,160 genes), “response to stimulus” (7,053 genes), and “biological regulation” (6,758 genes). In the molecular function category, composed of 19 functional groups, “binding” (11,702 genes) and “catalytic activity” (9,483 genes) were the most highly represented groups. In cellular component, “cell” (19,112 genes) “cell part” (19,056 genes) and “organelle” (15,537 genes) were the most represented groups.

### Differentially expressed genes (DEGs) involved in the heat-stress responses of Korean fir

**Figure 2:**
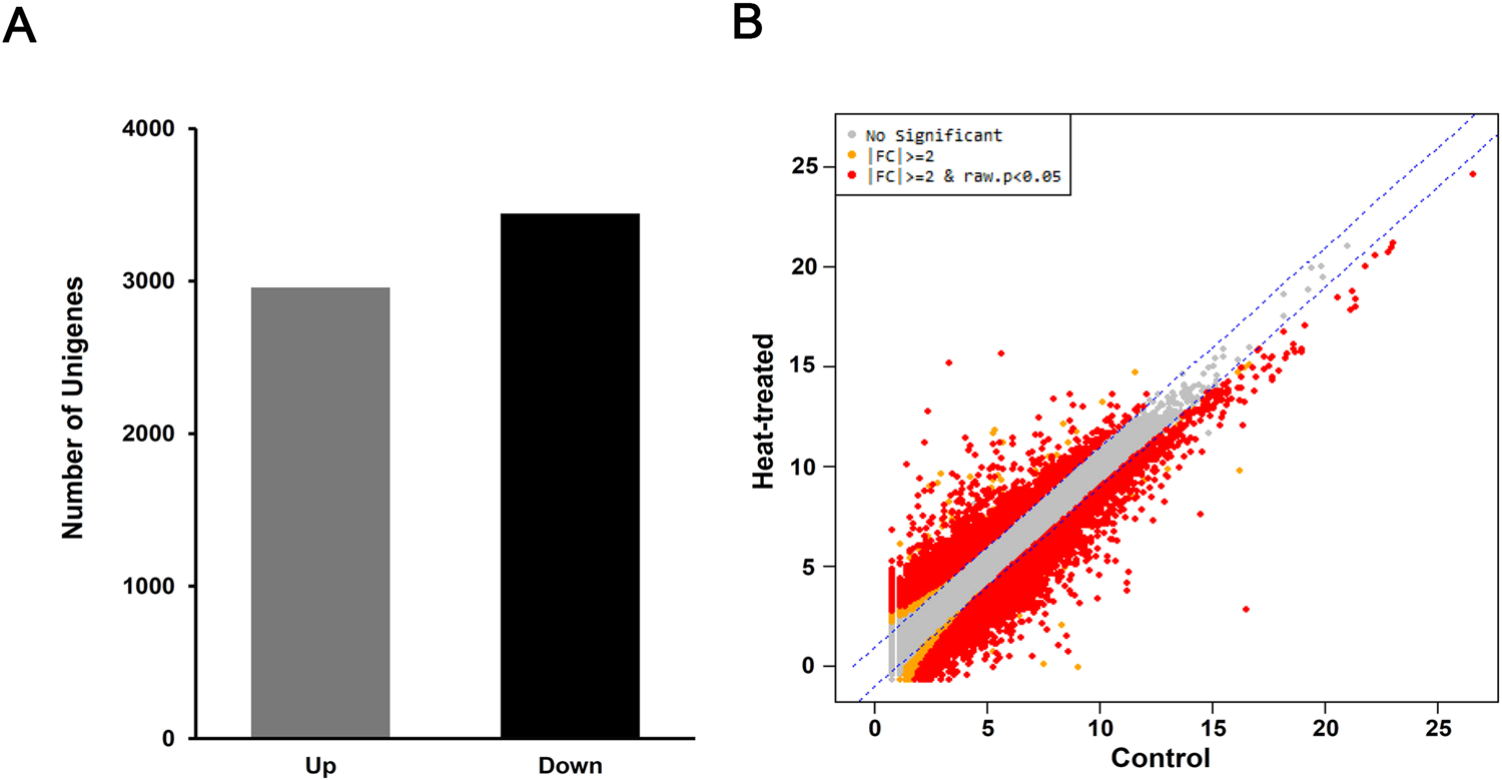
Distribution of differentially expressed *Abies koreana* unigenes in heat-treated samples compared with control conditions. (**A**) Distributions of up-regulated and down-regulated unigenes. The up-regulated and down-regulated unigenes indicate two fold change (FC ≥ 2) and P value < 0.05 in comparison with under control conditions. (**B**) Scatter plot of the normalised expression levels of all unigenes under control and heat-treated conditions. Each point represents the mean expression level of a gene under control and heat-treated conditions. Red dots represent differentially expressed genes between the conditions.

To identify potential heat-stress-responsive genes in Korean fir, the gene expression profiles were compared between control and heat-treated samples. For each gene of the assembly, the number of mapped reads was compared between the control and the heat-treated samples (Fig. 2). As a result, 6,401 were found to be DEGs (fold change ≥ 2, p < 0.05), with 2,958 up-regulated genes and 3,443 down-regulated genes in heat-treated sample compared (Supplemental Table S2). The distribution of transcript changes is shown in Fig. 2.

To investigate the biological roles of genes regulated by heat stress in Korean fir, we identified DEGs among the enriched GO terms, which were separated into the three main categories, biological processes, molecular functions, and cellular components (Supplemental Table S3). The top 20 most enriched functional groups are shown in Table 3. Among these, 11 functional groups (55%), including “cellular process”, “metabolic process”, “single-organism process”, “response to stimulus”, “biological regulation”, and “regulation of biological process”, were significantly enriched within the biological processes category. Two functional groups (10%), including “binding” and “catalytic activity” were significantly enriched within the molecular functions category, and seven functional groups (35%) “cell”, “cell part”, “organelle”, “membrane”, “organelle part”, “membrane part” and “macromolecular complex”, were significantly enriched within the cellular component category. Thus, changes in the biological processes category appear to be of great significance in the response to heat stress in Korean fir.

**Table 3:** Top 20 most enriched functional groups in the gene ontology categories.

Functional groups	GO-id	Unigene number
Biological process		
cellular process	GO:0009987	2578
metabolic process	GO:0008152	2424
single-organism process	GO:0044699	2294
response to stimulus	GO:0050896	1305
biological regulation	GO:0065007	1212
regulation of biological process	GO:0050789	1121
localization	GO:0051179	782
cellular component organization or biogenesis	GO:0071840	747
developmental process	GO:0032502	676
multicellular organismal process	GO:0032501	659
signaling	GO:0023052	500
Molecular functions		
binding	GO:0005488	2102
catalytic activity	GO:0003824	1815
Cellular components		
cell	GO:0005623	3215
cell part	GO:0044464	3199
organelle	GO:0043226	2435
membrane	GO:0016020	1244
organelle part	GO:0044422	847
membrane part	GO:0044425	760
macromolecular complex	GO:0032991	414

### Identification of transcription factors (TFs) involved in heat stress

**Figure 3:**
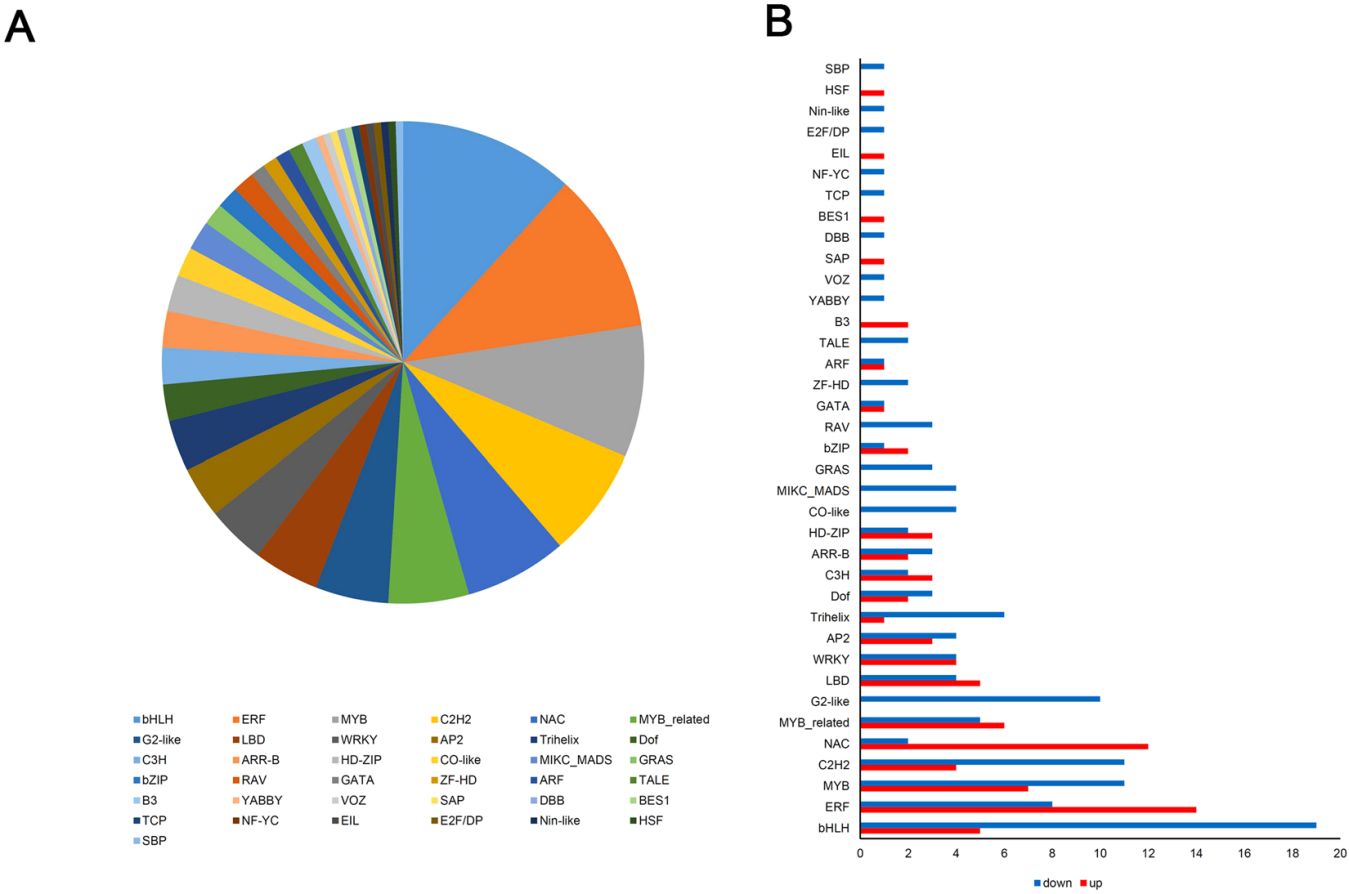
Family distribution of the transcription factors in the Korean fir transcriptome. (**A**) The number of each transcription factor family’s member. (**B**) Up- or down-regulated transcripts from every transcription factor family involved in transcription.

The transcriptional regulation of heat stress has been widely documented in model plants^21^. To identify the TFs involved in heat-stress responses, we surveyed the putative TFs that were differentially expressed in Korean fir under heat stress. The TFs in this study were compared with *P. abies* transcriptome sequences obtained from publicly available datasets (E-value < 1e^−10^). A total of 204 TFs (81 up-regulated and 123 down-regulated) showed more than twofold stronger signals in the heat treated sample than in the control (Supplemental Table S4 and Fig. 3). Most abundantly represented, we identified the gene family involved in the basic helix-loop-helix family (bHLH), followed by ethylene-responsive element-binding factors (ERF), MYB, C2H2 family, and the NAC family. Of these TF families, bHLH, including 24 unigenes (5 up- and 19 down-regulated), ERF, including 22 unigenes (14 up- and 8 down-regulated), and MYB, including 18 unigenes (7 up- and 11 down-regulated), were the three most enriched TF families. Almost of the NAC TF family unigenes (12 of 14) were up-regulated under heat-treated conditions (Fig. 3). Taken together, the analysis provided a profound illustration of the roles of TFs under heat stress.

### Identification of heat shock proteins (Hsps)

To begin to elucidate the molecular basis of heat-stress tolerance in Korean fir, we sought to identify sequences in the transcriptome that encoded authentic Hsps. Based on sequence conservation (E-value < 1e^−10^), we identified 189 putative Hsp genes (Supplemental Table S5). Most of these *Hsps* were significantly up-regulated during the heat treatment (Supplemental Table S5). Of these unigenes, Trans Decoder identified 30 complete open reading frames with putative start and stop codons (Table 4).

**Table 4:** Differentially expressed *Abies koreana* transcripts identified as heat shock protein (Hsp) families.

Classification	Contigs	Annotation	Fold change
Hsp90	c212080_g4_i1	HSP90_0281|Vitis vinifera	4.66
	c188934_g1_i1	HSP90_0278|Vitis vinifera	3.82
	c273438_g1_i1	HSP90_0266|Vitis vinifera	3.25
	c218689_g1_i1	HSP90_0266|Vitis vinifera	2.18
	c218750_g1_i1	HSP90_0266|Vitis vinifera	2.17
	c218389_g1_i1	HSP90_0266|Vitis vinifera	2.10
Hsp70	c151899_g1_i2	HSP70_1149|Vitis vinifera	7.20
	c149565_g1_i1	HSP70_1146|Vigna radiata	5.80
	c210065_g1_i1	HSP70_1095|Solanum lycopersicum	3.96
	c201565_g1_i2	HSP70_1078|Ricinus communis	3.48
	c194240_g1_i1	HSP70_1154|Vitis vinifera	2.70
	c207016_g1_i1	HSP70_0928|Arabidopsis thaliana	2.61
	c203374_g1_i1	HSP70_1077|Ricinus communis	2.29
	c149639_g1_i1	HSP70_1102|Sorghum bicolor	−5.06
Hsp60	c199303_g3_i1	HSP60_1249|Vitis vinifera PN40024	18.04
	c208087_g1_i3	HSP60_1095|Oryza sativa Indica Group	7.49
	c195910_g3_i3	HSP60_1095|Oryza sativa Indica Group	7.39
	c40393_g1_i1	HSP60_1095|Oryza sativa Indica Group	6.19
	c195910_g4_i1	HSP60_1095|Oryza sativa Indica Group	6.10
	c208445_g2_i2	HSP60_1095|Oryza sativa Indica Group	5.46
	c214017_g2_i1	HSP60_1044|Arabidopsis thaliana	4.41
	c196455_g1_i1	HSP60_1171|Ricinus communis	4.30
	c208445_g3_i1	HSP60_1095|Oryza sativa Indica Group	2.37
	c175049_g1_i1	HSP60_1249|Vitis vinifera PN40024	−2.94
	c194574_g1_i2	HSP60_1095|Oryza sativa Indica Group	−3.28
	c195155_g1_i1	HSP60_1249|Vitis vinifera PN40024	−3.74
	c178445_g1_i1	HSP60_1249|Vitis vinifera PN40024	−4.30
sHsp	c207145_g1_i1	sHsp_0659|Physcomitrella patens subsp. patens	4.91
	c201988_g1_i1	sHsp_0819|Vitis vinifera PN40024	4.85
	c213666_g1_i3	sHsp_0673|Ricinus communis	3.17
Average expression levels			3.39

### Validation of DEGs using qRT–PCR

**Figure 4:**
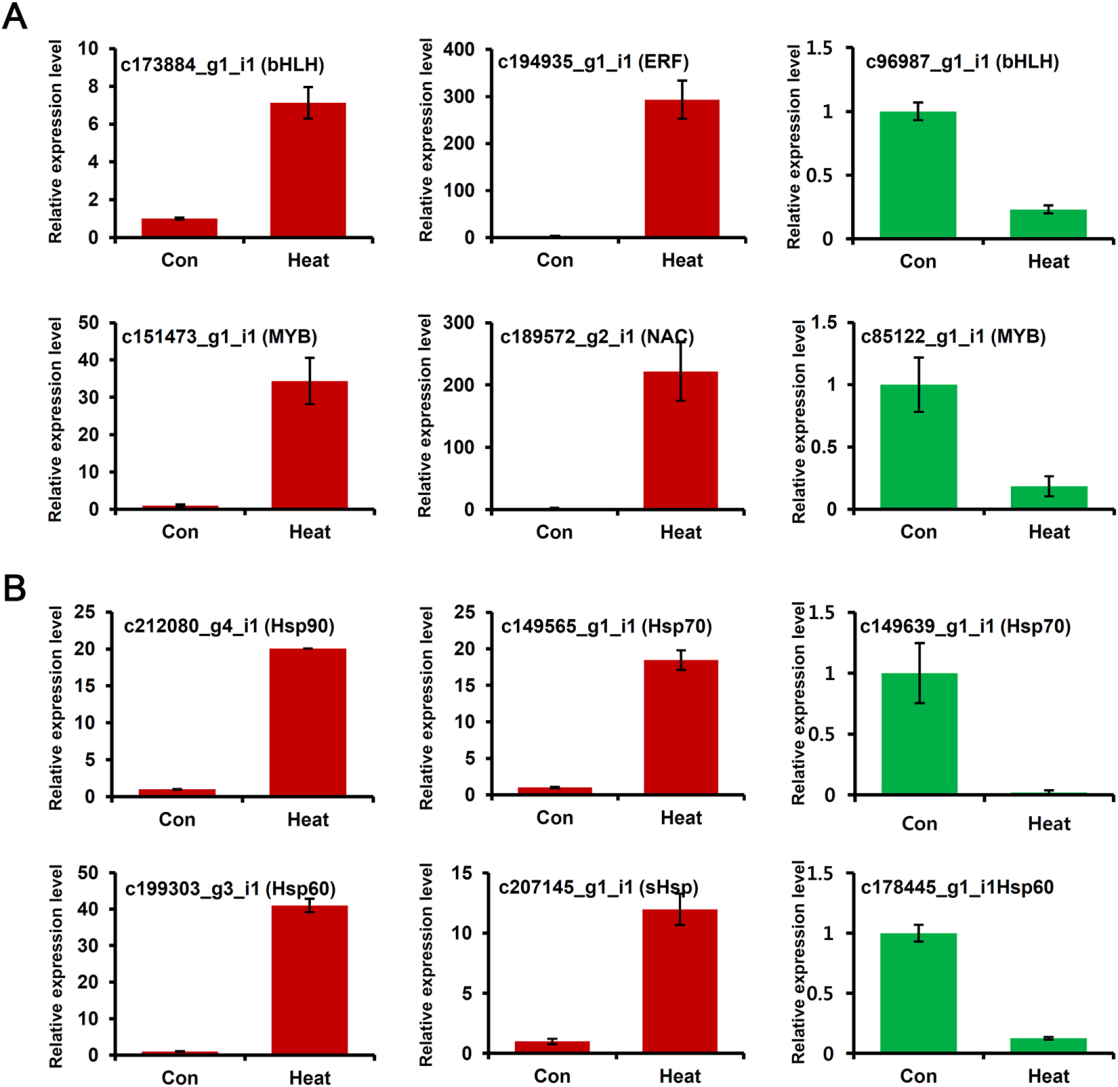
qRT-PCR expression analyses of 6 transcription factor and 6 heat shock proteins in response to heat stress. qRT-PCR was performed to validate the results of the RNA sequencing analysis using cDNAs prepared from 3-year-old needles of Korean fir exposed for 21-day to control (22 °C) or heat-treatment (30 °C) conditions. Error bars denote standard errors of biological replicates. Expression values of each gene are normalised against the expression of *Actin*^18^.

To confirm the accuracy of the RNA-seq results, 12 DEGs were randomly selected for a qRT–PCR-based comparison of their expression levels between the control and heat-treated samples (Fig. 4). The primer sequences are listed in Supplemental Table S6. All 12 DEGs in the control and heat-treated samples showed the same expression patterns in the qRT–PCR (Fig. 4). The unigenes included six putative heat-related TFs. The heat treatment up-regulated c173884_g1_i1 (bHLH), c194935_g1_i1 (ERF), c151473_g1_i1 (MYB), and c189572_g2_i1 (NAC) and down-regulated c96987_g1_i1 (bHLH), and c85122_g1_i1 (MYB) (Fig. 4A). The remaining six unigenes encoded Hsps. The expression levels of c212080_g4_i1 (Hsp90), c149565_g1_i1 (Hsp70), c199303_g3_i1 (Hsp60), and c207145_g1_i1 (sHsp) were up-regulated by heat-treatment (Fig. 4B), while the expression levels of c149639_g1_i1 (Hsp70), and c178445_g1_i1 (Hsp60) were down-regulated by heat-treatment (Fig. 4B). This independent evaluation confirmed the reliability of the RNA-seq data. Also, this strongly supported the involvement of these 12 unigenes in responses to heat.

**Figure 5:**
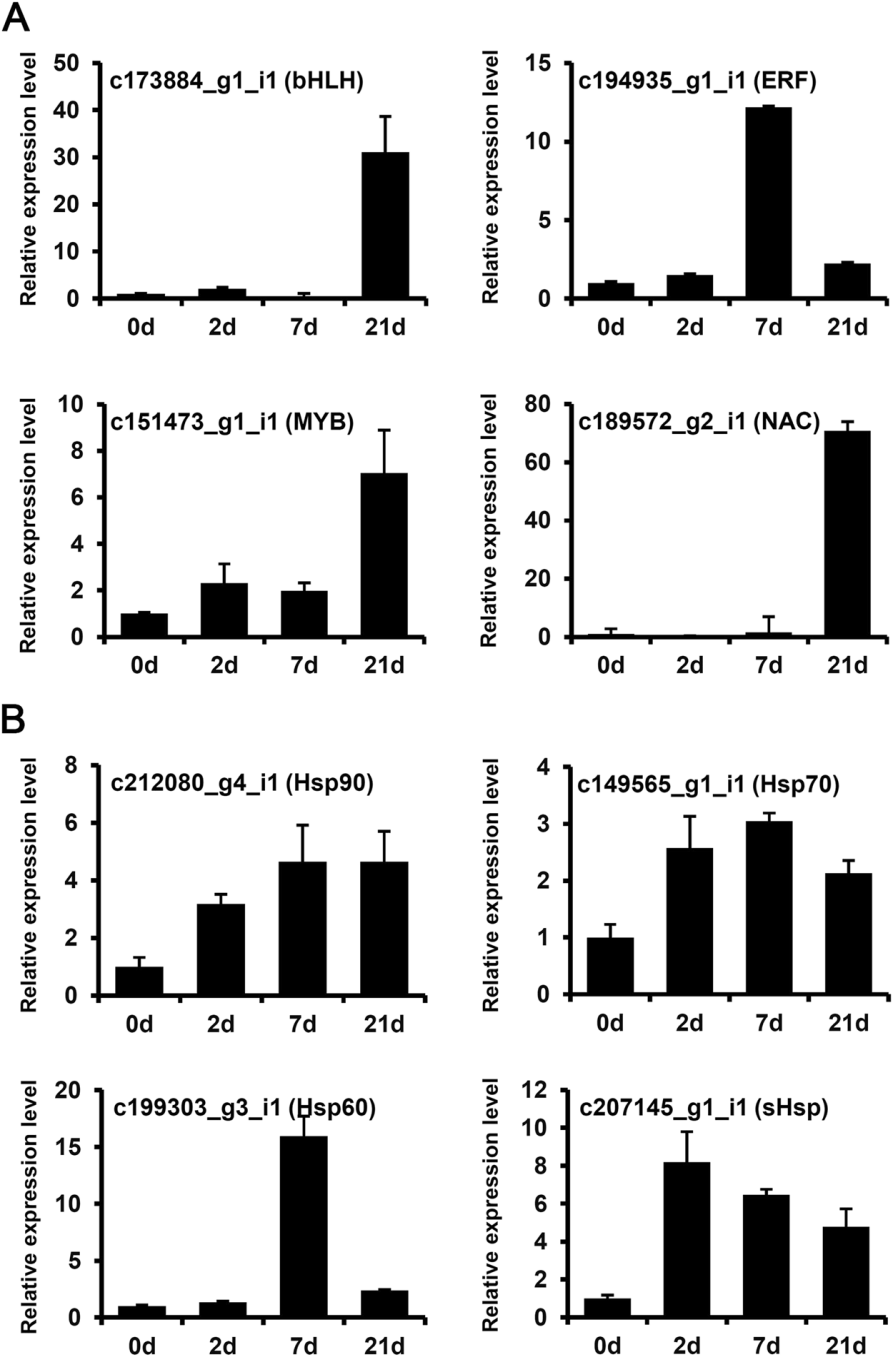
qRT-PCR expression analysis of 8 up-regulated genes at different time point in response to heat stress. Relative expression levels of 8 genes at 0, 2, 7 and 21-day after heat treatment (30 °C) were examined by qRT-PCR. Error bars denote standard errors of biological replicates. Expression values of each gene are normalised against the expression of *Actin*^18^.

Furthermore, eight up-regulated genes involved in heat tolerance were further investigated by qRT-PCR at different time points to confirm the expression profiles under heat stress in Korean fir (Fig. 5). Transcription factors ERF had the highest expression levels at 7-day, and bHLH, MYB and NAC had their highest expression levels at 21-day (Fig. 5A). The three Hsp genes (c212080_g4_i1, c149565_g1_i1 and c207145_g1_i1) showed similar fold changes at all of the time points (Fig. 5B). In most cases, the expression changes were significant after heat treatments (Fig. 5). Thus, these genes might be important in the response to heat stress in Korean fir.

## Discussion

In the absence of a whole genome sequence, RNA-seq provides an effective application tool for comprehensive studies of gene expression and the detection of novel transcripts associated with valuable traits^22^. In this study, we implemented a *de novo* RNA-seq technology to obtain insights into the transcriptomic responses induced by heat stress in Korean fir.

A whole-transcriptome analysis was performed in both heat-stressed and unstressed plants. For each sample, more than 160 M high-quality clean reads were obtained, which were *de novo* assembled into 406,207 contigs with an average length of 472.74 bp (Tables 1 and 2). These sequences produced longer contigs than those assembled in previous studies, such as blueberry (227 bp)^23^, *Gossypium aridum* (229 bp)^24^, *Hevea brasiliensis* (141 bp)^25^, *Paeonia ostii* (307 bp)^26^, pakchoi (343 bp)^27^ and sweet potato (202 bp)^28^.

Functional annotation and classification provide predicted information on cellular metabolic pathways and the condition-specific behaviour of gene expression. GO is an internationally standardised gene functional classification system that offers a dynamically-updated controlled vocabulary and a strictly defined structure to describe the properties of genes and their products in any organism^29^. Among the unigenes, 22,880 (54.40%) known proteins were assigned to GO classes. However, a large proportion of unigenes (45.60%) failed to match these databases owing to the paucity of gene information for genus *Abies*. According to the GO classification, “cellular process”, “binding”, and “cell” comprised the largest groups in the three main GO categories of biological processes, cellular components, and molecular functions, respectively (Fig. 1). Our GO classifications of the annotated unigenes provide a general gene expression profile signature for Korean fir (*A. koreana*) that will facilitate further studies not only in this species but also in other species of genus *Abies*.

Subsequently, we analysed the differentially expressed genes in the heat-treated and control samples. Under heat stress, the GO category of biological processes was enriched (Table 3). The large proportion of the terms were included the “cellular process”, “metabolic process”, and “single-organism process” (Table 3), indicating that wide-ranging changes in Korean fir gene expression levels occurred in response to heat treatment. These findings indicated significant chances in category of biological process as a response to heat stress. Additionally, many unigenes were over-represented as belonging to the category “response to stimulus” in the heat-treated sample (Table 3), and these unigenes represented the most important components directly involved in protecting plants from stress.

Apart from the list of unigenes that strongly responded to the changing environment (Fig. 2 and Supplemental Table S2), we focused on TFs, sequence-specific DNA-binding proteins that interact with *cis*-elements in the promoter regions of target genes and thus modulate gene expression. These TFs regulate gene transcription in response to biotic and abiotic stresses, such as cold, high temperatures, high salinity, drought, and pathogen attacks^7,10^. In our results, several TF families were identified as being involved in heat-stress responses, including bHLH, ERF, MYB, NAC, and C2H2 (Fig. 3).

The highest number of up-regulated genes are ERF family TFs, and an ERF coactivator gene is synergistically expressed with ERFs under heat stress^30^. The expressions of *AtERF53* and *ERF1* are induced by heat treatment in Arabidopsis and pakchoi, respectively^27,31^. The DREB2’s TF group belongs to the AP2/ERF family, and it has been characterised in the heat regulatory pathway^32^. The induced DREB2 functions to enhance heat tolerance in various plants^33,34^. Other TFs, including bHLH, MYB, and C2H2 families, were also altered during heat treatments, and many members of these gene families function in heat tolerance^35,36^. The *ERF*, *bHLH*, *MYB*, and *C2H2* pathways are conserved in Korean fir’s responses to heat stress. The plant-specific NAC TF family has been implicated in the regulation of diverse processes, including hormone signalling, defence, and stress tolerance. NAC TFs in plants are mainly involved in osmotic stresses, including drought and high salinity^37^. However, some NACs (RD26) function in response to cold stress^38^. Morishita *et al*.^39^ also reported that ANAC078 in the NAC group TIP is responsive to a combination of high light and heat stress. We found 14 genes encoding NAC TF domains, and almost the genes were up-regulated and showed significant expression levels by RNA-seq and qRT–PCR (Table S4, Figs 4 and 5). The results can provide insights that will explain critical functions of the NAC family of genes in the heat responses of Korean fir.

Heat shock factor (HSF) TFs are key regulators involved in responses to heat stress^40^. Most plant HSFs are, mostly induced, regulated by heat stress. Among 23 rice *OsHsf* genes, 16 *OsHsfs* were up-regulated, however, *OsHsfC1a* was noted to be down-regulated by heat stress^41^. Similarly, many HSF genes from different plant species, such as Arabidopsis, cotton, maize, apple and pepper showed up- and down-regulation by heat stress^42^. However, we found only one transcriptional HSF, which was up-regulated in our data set (Fig. 3 and Supplemental Table S4). The small number of expressed HSFs (Fig. 3) might indicate that the heat-response pathway responded, at least partially, in an HSF-independent manner in the signalling network in Korean fir. Future studies should reveal such a manner of regulation.

In addition, several seemingly novel TF families responding to heat shock (ARR-B, AP2, C3H, and G2-like; Fig. 3) were also identified. Homologs in other plant species have not yet been reported in the response to heat stress, suggesting that the behaviour of these genes might be *Abies* species specific, making them attractive targets for further functional characterisation. The findings should expedite studies focusing on the interactions of different TFs in the regulation of heat stress. Thus, our analysis combines considerably conserved responses in addition to seemingly novel, possibly conifer specific, components involved in the heat-stress response mechanisms across plant species.

The analyses of transcriptome profiles in plants after heat treatment have amply documented the central role of the various HSP families in heat-stress responses^30,43,44^. Hsp families, including Hsp100, Hsp90, Hsp70, Hsp60, and small Hsps, are involve in folding and assembling proteins, maintaining protein stabilisation, activating proteins, and degrading proteins in many normal cellular processes and under stress conditions^45^. In our study, the expression levels of most Hsp genes in Korean fir were up-regulated after heat stress (Table 4, Figs 4 and 5). Thus, inductions of Hsps are critical for acclimating to heat stress in this species.

This first comprehensive transcriptomic analysis of Korean fir provides a valuable genomic resource for further studies, including as well in other *Abies* species. Additionally, our study provides new insights into heat-stress adaptation; it will facilitate further studies on Korean fir genes, their regulation and their functions. Our study represents a fully characterised transcriptome and provides valuable resources for genomic studies in Korean fir under heat stress and beyond.

## Methods

### Plant material and treatments

Korean fir (*Abies koreana* Wilson) seeds were collected from Mount Halla on Jeju Island, Korea (33° 13–36´N, 126° 12–57´E). Seeds were sown in seedling trays with soil after breaking dormancy at 4 °C for three months. A single 1-year-old seedling was transplanted into each pot filled with the same soil. Plants were grown in a greenhouse under natural sunlight conditions.

The 3-year-old seedlings were exposed to normal growth conditions (22 °C) and heat stress (30 °C) under 16 h of 100 µE s^-1^ m^-2^ light and 8 h dark. For RNA-sequencing, all of the needles were harvested after a 21-day heat treatment at which time controls were harvested as well. Three biological replicates were harvested. For the quantitative RT-PCR (qRT-PCR) at different time points, plants were harvested at 0, 2, 7 or 21-days after the treatment. All of the needles were frozen in liquid nitrogen and stored at −80 °C for RNA extraction.

### Library preparation and RNA-sequencing

RNA samples were extracted from the needles of 21-day heat-treated and control plants. Total RNA was isolated using TRIzol reagent according to the manufacturer’s protocol (GibcoBRL, Cleveland, OH, USA). The RNA was analysed for quality and concentration using a 2100 Bioanalyzer (Agilent Technologies, Palo Alto, CA, USA). A total of 3 µg of RNA for each sample was used in library construction with the Truseq Stranded Total RNA sample Preparation Kit (Illumina, Inc. San Diego, CA, USA) per the manufacturer’s instructions. Briefly, mRNA was enriched using magnetic beads containing poly-T molecules. Following purification, the enriched mRNA was broken into small fragments. Random oligonucleotides and SuperScript II were used to synthesize the first-strand cDNA. The second-strand cDNA was subsequently synthesized using DNA Polymerase I and RNase H. Finally, end repair was carried out on these cDNA fragments, and they were ligated with Illumina adapters. Libraries were amplified using PCR according to Illumina guidelines. Libraries with insert sizes from 290 to 330 bp were constructed, and sequenced using the Illumina HiSeq. 2500 to generate 101-bp paired-end reads.

### *De novo* transcriptome assembly and annotation

The raw reads were first cleaned by filtering out adaptor sequences and low-quantity reads using Trimmomatic (version 0.32). Trimmed reads were combined across all conditions and *de novo* assembled with Trinity software (version R20140717)^46^ using default settings to build a suitable set of reference contigs. Then, CD-HIT was used for further clustering with a 200-bp sequence length and 90% similarity cut-off values to obtain non-redundant transcripts^47^. High-quality reads were mapped back to the assembled transcriptome sequences for validation. Reads were aligned using Bowtie with default parameters^48^. RSEM software was subsequently employed to calculate the abundance for each transcript, and filtered raw read count were retained for further processing^49^. These sequences were named as unigenes. Gene functions were annotated using BLASTX (BLAST 2.6.0+) based on the NCBI non-redundant protein sequences, GO and KEGG with default parameters. A functional enrichment analysis of unigenes using the GO categories molecular functions, biological processes, and cellular components was performed based on the protein sequence similarity in the GO database^29^. The number of genes assigned in each GO term was counted using WEGO website (http://wego.genomics.org.cn/).

### Identification of differentially expressed genes (DEGs)

We identified differentially expressed genes with DEseq.^50^ using per gene expected counts for each sample generated by RSEM^49^. A twofold change (FC ≥ 2) and P value < 0.05 were used to define the significant DEGs between treatment and control. GO enrichment analyses were performed using GO database and WEGO website.

### Transcription factors (TFs) and heat shock proteins (Hsps) analysis

To identify the putative TFs and decipher their roles during heat-stress responses, plant-specific TFs were downloaded from the Plant Transcription Factor Database (http://plntfdb.bio.uni-potsdam.de/v3.0/). A BLASTX algorithm-based search was performed with DEG sequences using the E-value cut off ≤ 1e^−10^ and classified unigenes according to the gene family’s information. Similarly, to identify canonical Hsps represented in our samples, DEG sequences were queried against a list of Hsp domain sequences from the HSRIP (http://pdslab.biochem.iisc.ernet.in/hspir) database. A stringent domain matching criteria of E-value ≤ 1e^−10^ was used to select Hsp domains. Following alignments, TransDecoder (http://transdecoder.sourceforge.net/) was used to predicate optimal open reading frame information.

### Quantitative RT-PCR (qRT-PCR)

To determine the reliability of the RNA-seq data, qRT-PCR was performed on the same RNA pools previously used for RNA-seq. Twelve DEGs, six from four groups having the largest TF transcript numbers and six DEGs from each group of Hsps, were randomly selected for qRT-PCR analysis. Furthermore, to verify the differential expression, qRT-PCR was performed on a new set of 3 replicates for each sample at different time points.

Total RNAs (1 µg) of each sample were reverse transcribed using a Power cDNA Synthesis Kit (Intron Biotech Inc., Sungnam, Korea). The specific primers used for qRT–PCR are listed in Supplemental Table S1. qRT–PCR was carried out on a Bio-Rad CFX qRT–PCR detection system (Bio-Rad Laboratories Inc., CA, USA) using iQ™ SYBR® Green supermix (Bio-Rad). The reaction was performed under the following conditions: 95 °C for 10 min, followed by 45 cycles of 95 °C for 10 s and 60 °C for 30 s. Their relative expression levels were calculated using the relative 2^−∆∆Ct^ method^51^. The qRT–PCR reactions were repeated in three biological and three technical replications.

## Electronic supplementary material

Supplementary dataset 1
